# The Dark Side of the Moon: A Good Adoption Rate Conceals the Unsolved Ethical Problem of Never-Adopted Dogs

**DOI:** 10.3390/ani15050670

**Published:** 2025-02-25

**Authors:** Sara Corsetti, Eugenia Natoli, Livia Malandrucco

**Affiliations:** 1 School of Agriculture and Environment, The University of Western Australia, Crawley, WA 6009, Australia; 2 Canile Sovrazonale, ASL Roma 3, 00148 Roma, Italy; enatoli@tiscali.it (E.N.); livia.malandrucco@aslroma3.it (L.M.)

**Keywords:** domestic dog, adoption, shelter, no-kill policy, laws, ethical implications

## Abstract

The no-kill policy for domestic dogs, adopted in Italy in 1991, reflects a growing interest in animal welfare and increases the chances of dogs being adopted and living with a family. However, unadoptable dogs must spend their entire lives in shelters. This paper reports the adoption numbers from the ‘Muratella’ shelter (Rome) during the periods 2012–2016 (first period) and 2018–2022 (second period), illustrating the outcomes of the no-kill policy in a large metropolitan area. Furthermore, it discusses the ethical implications after 34 years of implementing Italian laws on the management of free-ranging dogs. During the first period, 7888 dogs entered the shelter, of which 4247 (53.84%) were adopted and 307 (3.89%) were not adopted. Similarly, during the second period, 8853 dogs entered the shelter, of which 4251 (49.11%) were adopted and 278 (3.16%) remained in the shelter by the end of 2022. Furthermore, we report the fate of unadopted dogs by the end of 2024. Despite the success in adoptions, the small percentage of dogs condemned to lifelong shelter residence still represents a significant number and warrants ethical reflection.

## Simple Summary

Driven by society’s growing interest in animal welfare, nine European countries, including Italy, have adopted a no-kill policy for unowned domestic dogs. This approach increases the likelihood of dogs being adopted and living with a family. However, dogs deemed unadoptable are required to spend their entire lives in shelters. In countries where the no-kill policy is implemented by law, several challenges arise, including managing the costs associated with the large number of dogs in shelters and addressing the ethical dilemma of keeping non-adoptable dogs confined for life. While adoption rates are generally successful, the small percentage of dogs condemned to lifelong shelter residence still represents a significant number and poses important ethical concerns.

## 1. Introduction

Nowadays, there has been growing interest in animal welfare within the scientific community [1]. Initially, attention was primarily focused on laboratory and farm animals, with the aim of ensuring reliable research outcomes and increased productivity [2,3,4]. However, this focus has since expanded to include wildlife and companion animals, driven by conservation, economic, and ethical considerations.

Among companion animals, dogs are considered the quintessential pets. Despite their increasing popularity as household companions, a significant number of dogs still lack a permanent home; it is sufficient to say that approximately 80% of the global dog population consists of free-ranging dogs that mate and move freely within their territories [5]. However, it is important to emphasise that free village dogs in many countries (e.g., India and Africa) must be distinguished from free-ranging dogs in Westernised countries, as the human interest and management of these animals differ significantly. In fact, to address this issue in Westernised countries, human societies have established facilities such as shelters to house free-ranging animals ensuring the safety of both humans and animals [6]. Initially, shelters were primarily intended to remove unwanted animals from streets and public areas. However, especially in countries where the ‘no-kill policy’ is enforced by law, their current focus has shifted toward rehoming or returning as many animals as possible. In shelters, dogs are provided with food, water, shelter, and veterinary care and, nowadays, there is an increasing emphasis on enhancing their well-being by promoting positive welfare states and minimizing negative ones [7]. Despite these advancements, shelters remain inherently stressful environments for the animals housed within them [8,9,10,11,12,13,14,15,16,17]. Dogs are often exposed to unfamiliar surroundings, spatial and social confinement, excessive noise, and, in some cases, separation from a human attachment figure [12,18].

Ensuring the highest possible level of animal welfare is particularly important in some countries, such as Italy, where a no-kill policy has been implemented. Italy was the first country in the world to establish a no-kill policy with Law no. 281 (in 1991). Recently, Italy has been joined by other countries, such as Croatia and Portugal, in implementing a no-kill policy that allows euthanasia only for dogs proven to be dangerous or suffering from an incurable disease, based on veterinarians’ professional diagnosis, in contrast to other European states. As a result of this law, thousands of healthy dogs are no longer euthanised, leading shelters to face a higher-density dog population than ever. Shelter staff have focused their efforts on increasing adoptions, which has led to good results with a growing percentage of animals finding new homes outside the shelter (e.g., [19]). However, there is another side to this issue: many dogs remain unadopted and spend their entire lives confined to cages, sometimes with no social interactions—either inter- or intra-specific—especially if they exhibit some aggressive behaviour or simply belong to breeds, such as molossoids, that are perceived as potentially dangerous in the collective imagination.

Thus, the question is: when can a dog’s future be considered worth living?

The aim of this paper is to evaluate the length of stay of dogs at the Municipal Sovrazonal Dog Shelter “Muratella” in Rome and assess how age, sex, body size, breed, and coat colour influenced adoption trends over two recent five-year periods: from 2012 to 2016 (first period) and from 2018 to 2022 (second period). The numbers that resulted from this evaluation are necessary to reflect on and provoke an ethical debate on the thousands of dogs that remain unadopted and are forced to spend their entire lives in confinement, ostensibly for the benefit of humane societies [19]. While providing solutions is beyond the scope of this article, a more in-depth discussion should consider the wider impacts and consequences of implementing a no-kill policy. We hope that this article will contribute to a more complete and honest cost–benefit analysis of this policy.

## 2. Materials and Methods

### 2.1. Animals and Housing

The cohort included 7888 dogs (4458 males and 3430 females) that entered the Municipal Sovrazonal Dog Shelter ‘Muratella’ in Rome (Italy) in the first period (from 1 January 2012 to 31 December 2016), and 8656 dogs (4976 males and 3683 females) that entered in the second period (from 1 January 2018 to 31 December 2022). The year 2017 was excluded because in that year there was a change of management between the entities entrusted by the municipality of Rome.

Dogs were housed alone or in pairs in cages (4 m^2^) with an indoor and an outdoor area. Outside the cages, there was a fenced zone where dogs were free to move for 10–15 min twice a day. During this time, the cage was cleaned by the shelter staff. Individuals who did not show signs of aggressive behaviour were taken out of a cage for a walk by shelter staff or volunteers. Dogs were fed twice a day both with wet and dry high-quality food.

Upon entering the shelter, each dog underwent a clinical examination before being assigned to a cage. During this examination, a veterinarian performed routine checks (i.e., temperature control, visual physical examination of the ears and mouth), treated the animal for ecto- and endo-parasites, and collected a blood sample in order to monitor *Leishmania infantum*. Each dog was also vaccinated against distemper, Rubbarth’s hepatitis, leptospirosis, and diseases caused by paramyxovirus and parvovirus (gastroenteritis). A form of behavioural assessment also took place during the clinical intake examination of newly admitted dogs, although no formal protocol was in place. Intuitively, handling is required during a veterinary visit, allowing for an initial evaluation of the dog’s behaviour. Some dogs remained calm when handled, while others reacted with fear or aggression. However, these reactions often change over time. In fact, many dogs entering the shelter had been wandering for days or even months—they may have been hungry, frightened, injured, ill, infested with endo- and ectoparasites, or even victims of abuse. Under such circumstances, the clinical intake examination provided little meaningful insight into a dog’s true behavioural tendencies. For this reason, implementing a formal behavioural assessment at intake would likely be both ineffective and unnecessary.

Most of the dogs housed at the Muratella shelter contracted some infectious disease during their stay. However, these were generally not severe, as the dogs were vaccinated against the most serious diseases during the clinical examination upon arrival. Therefore, quantifying how many dogs ‘fell ill’ during the selected periods would not yield particularly informative data. Instead, we reported the more relevant data of dogs that entered already sick or injured, along with their fate (recovery, death, euthanasia, adoption, or other).

### 2.2. Dog Shelter Organisations

The Muratella shelter operates as both a sanitary dog shelter of the Rome 3 Local Health Unit Veterinary Services (https://www.aslroma3.it/dipartimenti/dipartimento-di-prevenzione/uosd-canile-sovrazonale-e-controllo-del-randagismo/, accessed on 22 February 2025) and long-term public shelter hosting the dogs belonging to the municipality of Rome (https://www.canilidiroma.it/index.html, accessed on 22 February 2025) according to the legislative provisions of the Lazio Regional Law Nr. 34 of 21 Ottobre 1997, and the Annex 1 to the Lazio Region Resolution Nr.43 of 29 January 2010.

All dogs, when they enter the shelter, are under the jurisdiction of the Rome 3 Local Health Unit Veterinary Service, including those that appear healthy after the entrance examination. After a certain period of time, which can vary from a few days up to a few months, depending on the reasons for the dog’s entry and its health condition, the dog is neutered and transferred under the jurisdiction of the municipality of Rome, always in the same place but housed in different buildings. Once the dog has been transferred, the Local Health Unit operators no longer have direct access to its data. Responsibility for decisions regarding the sanitary aspects of the dogs is divided. Upon entry, and as long as the dog falls under the jurisdiction of the Local Health Unit Rome 3, the unit’s veterinarians are responsible for it. However, once the dog is transferred to the municipal shelter, responsibility shifts to the municipal veterinarians. The shelter managers are responsible for adoptions. Private citizens also have their share of responsibility, since such a high number of adoptions must be matched by a high level of responsible adoptions, and the data presented here show that this has not always been the case. However, instances of irresponsible management and failed adoptions have remained quite low.

### 2.3. Statistical Analyses

We performed a Chi-square test to compare the adoption rates of all dogs entering the shelter between the two five-year periods.

A Generalised Linear Model (GLMM) was conducted on dogs entering the shelter during the first period and the second period, excluding those redeemed or transferred to another shelter (resulting in 5105 out of 7888 and 4834 out of 8656 dogs analysed, respectively) to assess if sex, breed, body size, coat colour, and age at adoption influenced the possibility of a dog being adopted. We did not have detailed information about the coat colour of dogs entering the shelter in the first period, so this variable was excluded from the model.

The Kaplan–Meier estimates were computed to assess how the sex, age, size, and breed of dogs affected the timing of their adoption.

All the analyses were conducted using the IBM SPSS software (Version 28.0. Armonk, NY, USA: IBM Corp.).

## 3. Results

### 3.1. General Results

A total of 7888 dogs entered the shelter during the first period, compared to 8656 dogs in the second. Details on the sex, breed, body size, and age class of the dogs that entered the shelter are presented in Table 1.

**Figure 1:**
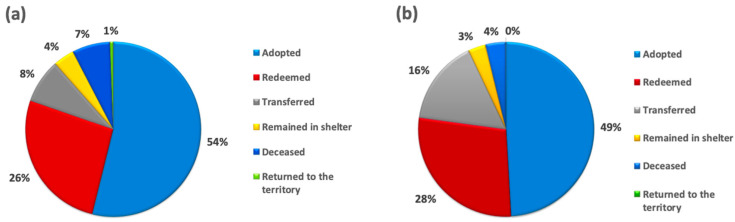
Fates of dogs entering the ‘Muratella’ shelter during the first period (**a**) and during the second period (**b**), along with their respective percentages: redeemed, adopted, still in the shelter, transferred, deceased (including natural causes and euthanasia), or returned to the territory.

During the first period, of the 7888 dogs that entered the shelter, 2088 (26.47%) were redeemed by the owners (data on the number of dogs without microchips are not available in the shelter database); 4247 (53.84%) were adopted (data on the number of adoptions failed are not available in the shelter database); 647 (8.20%) were transferred to another shelter offering similar conditions and management run by the Rome municipality because of the lack of space at Muratella; 551 (6.99%) passed away (including natural causes and euthanasia); 48 (0.61%) were returned to the territory in compliance with national and regional laws; and 307 (3.89%) remained in the shelter (Figure 1a). Similarly, in the second period, of the 8656 dogs, 2428 (28.05%) were redeemed by their owners (including 792 (32.62%) found without a microchip); 4251 (49.11%) were adopted (including 243 (5.72%) failed adoptions); 1369 (15.82%) were transferred to another shelter; 324 (3.74%) passed away (including natural causes and euthanasia); 6 (0.07%) were returned to the territory in compliance with national and regional laws; and 278 (3.21%) remained in the shelter (Figure 1b).

Excluding redeemed and transferred dogs, the shelter housed a total of 5105 dogs in the first period and 4853 dogs in the second one, whose fate could potentially have been adoption, death, or remaining in the shelter. The adoption percentages for each category (sex, breed, and body size class) are detailed in Table 2.

The results of the chi-square test, applied to dogs reported in Table 2, support the hypothesis that the number of adoptions is dependent on the number of dogs entered in each five-year period (X^2^ = 38.54, df = 1, *p* < 0.0001). Consequently, the adoption rate was significantly higher in the second five-year period than in the first.

**Figure 2:**
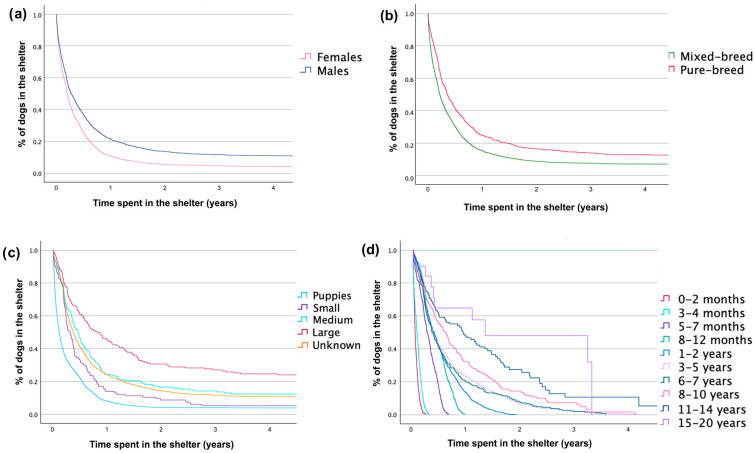
Cumulative permanence for dogs in the shelter ‘Muratella’ in Rome in the first period: (**a**) females (in pink) compared to males (in blue); (**b**) mixed-breed dogs (in green) compared to pure-breed derived dogs (in red); (**c**) puppies (size undetermined) (in light blue) compared to small (in purple), medium (in light green), and large (in red) size dogs; and (**d**) 0–2 months years old (in red) compared to 3–4 months (in azure), 5–7 months (in dark purple), 8–12 months (in light green), 1–2 years (in light blue), 3–5 years (in lilac), 6–7 years (in dark green), 8–10 years (in pink), 11–14 years (in dark blue), and 15–20 years old (in purple) dogs. A vertical drop in the curves indicates an event of adoption.

Sex, dog size, and age at adoption influenced the adoption rate for dogs entered in the first period (GLMM, Table 3, Figure 2).

The Omnibus test suggested that the model was highly significant in comparison to the null model (X^2^ likelihood ratio = 21,009.250, df = 16, *p*-value ≤ 0.001).

**Figure 3:**
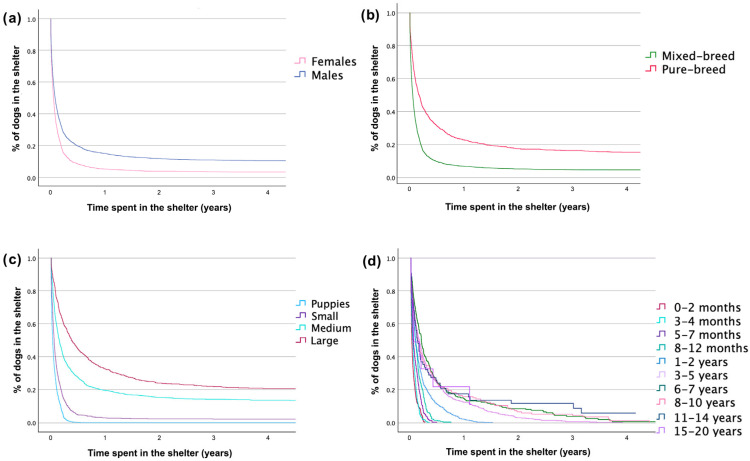
Cumulative permanence for dogs in the shelter ‘Muratella’ in Rome in the second period: (**a**) females (in pink) compared to males (in blue); (**b**) mixed-breed dogs (in green) compared to pure-breed derived dogs (in red); (**c**) puppies (size undetermined) (in light blue) compared to small (in purple), medium (in light green), and large (in red) size dogs; and (**d**) 0–2 months years old (in red) compared to 3–4 months (in azure), 5–7 months (in dark purple), 8–12 months (in light green), 1–2 years (in light blue), 3–5 years (in lilac), 6–7 years (in dark green), 8–10 years (in pink), 11–14 years (in dark blue), and 15–20 years old (in purple) dogs. A vertical drop in the curves indicates an event of adoption.

Similarly, sex, breed, dog size, and age at adoption, but not colour of the coat, influenced the adoption rate for dogs entered in the second period (GLMM, Table 4; Figure 3).

The Omnibus test suggests that the model is highly significant in comparison to the null model (X^2^ likelihood ratio = 21,007.623, df = 15, *p*-value ≤ 0.001).

In the first period, mixed-breed females less than two months of age experienced the shortest average shelter stays, remaining just 13.24 days. However, mixed-breed males older than two months but still very young endured the longest stays in the shelter, averaging 840.60 days, despite entering the shelter at less than a year old. Surprisingly, their stay length exceeded even that of much older large-sized dogs (11 to 14 years), which spent an average of 588.67 days (males) and 504 days (females) in the shelter, even when they were purebred.

### 3.2. Special Adoptions

Among the remarkable stories of this period are the so-called “special adoptions”. For instance, an 8-year-old Rottweiler found as a free-ranging dog without a microchip spent three years in the shelter before being adopted at the age of 11. Similarly, a 9-year-old male Abruzzese Maremma Sheepdog, once a “neighbourhood dog” (a status permitted by Lazio law), was taken to the shelter when he became ill and could no longer remain on the streets. He found a new home in just 27 days. Another touching example is an 11-year-old female German Shepherd, rescued with injuries, who was adopted after only 32 days. A particularly moving case involved a mixed-breed female who entered the shelter at just two months old and waited over three years—1115 days—before finding her forever home.

During the second period, small-sized purebred females aged three to four months had the shortest stays, often leaving the shelter within 24 h. In stark contrast, much older large-sized male purebred dogs (8 to 10 years old) faced the longest waits, with an average stay of 854.67 days.

Even during this timeframe, “special adoptions” stood out. One notable case was a male German Shepherd with a history of biting, who entered the shelter at over 6 years of age. Despite his challenges, he was finally adopted after spending 1587 days in the shelter.

### 3.3. Initial Health Status and Subsequent Outcomes of Dogs in the First and Second Period

Several dogs entered the shelter already injured or sick and received treatment, with varying outcomes depending on factors such as age, severity of injuries, and other variables (Table 5).

### 3.4. Non-Adopted Dogs

As expected, on the one hand, most of the unadopted dogs were large-sized, mixed-breed male dogs (Table 6), followed by molossoids which showed the highest percentage of biters (Table 7). On the other hand, an additional 48.53% of dogs were adopted by 31 December 2024 in the first period, including 4.70% classified as biters, while in the second period, 29.50% found a home, with 9.76% falling into the same category of biters (Table 8).

## 4. Discussion

### 4.1. Dog Management

Our findings align with the suggestions made by Natoli et al. in 2019 [19]. Following the introduction of Italy’s no-kill policy law for unowned free-ranging dogs and cats in 1991 (National Italian Law no. 281/1991: Legge quadro in materia di animali di affezione e prevenzione del randagismo), the role of shelters has become pivotal in managing captured free-ranging dogs. Over the years, the “Muratella” dog shelter has emerged as a central hub for dog management in the Rome area, fostering collaboration between municipal and regional authorities, private entities, and animal welfare volunteer associations. This synergistic approach aims to maximise the efficiency and effectiveness of the dog relocation system, and was successful. Adoption rates have been remarkably high: in the first period, 7888 dogs entered the shelter, of which 4247 (53.84%) were adopted. Similarly, during the second period, 8656 dogs were taken in, and 4251 (49.11%) found new homes.

Although certain categories of dogs, such as puppies, are more likely to be adopted than others (i.e., large-size dogs), dogs from any category still have a chance of finding a new home. This claim is supported by the results presented in this article regarding exceptional adoptions. While the term ‘exceptional’ inherently suggests that these cases are not the norm, these data remain valuable in the ethical debate, as they offer a glimpse of hope. Additionally, in the first five-year period (2012–2016), 2088 dogs (26.47%) were reclaimed by the owners. Similarly, during the 2018–2022 period, 2428 dogs (28%) were also reunited with their owners. Furthermore, since 792 dogs (32.62%) of the redeemed dogs were found without a microchip, one was implanted before returning the animals to the owners; all these actions highlight the valuable service the shelter provides to the community. Last but not least, from the point of view of dog welfare, one must consider the service the shelter performs in recovering free-ranging animals in a miserable condition (injured and/or sick) that, in addition to suffering, pose a danger to public health in terms of road accidents caused and the spread of diseases. Some of them recovered and were finally adopted.

As stressed in other studies [19], females have a higher adoption rate than males, and small-sized dogs are adopted more readily than medium- or large-sized ones. Coat colour did not influence adoption outcomes as demonstrated in other studies, further disproving the myth about the “Black Dog Syndrome” [20,21]. However, physical appearance plays a role in adoption, as reflected in the percentage of unadopted mixed-breed dogs, but it appears to be less significant than dangerous behaviour (see below).

Contrary to expectations, even older dogs were adopted and left the shelter, although adoption rates declined to around 50% for higher age groups. This success can be attributed to the shelter’s efforts, particularly those of volunteer associations, in promoting so-called ‘Adoptions of the heart’. These initiatives aim to find homes for elderly dogs, allowing them to spend their final years in a loving environment.

The improvement in the physical condition of injured and/or sick dogs rescued from the streets is clearly shown in Table 5. These results give rise to several reflections. Italian law no. 281/1991 enforces euthanasia only in cases of ‘incurable diseases and/or proven dangerousness’ of the animal. Setting aside the latter for now, let us focus on the former. More than 33 years after the law was enacted, the healthcare landscape has evolved, raising issues of definition. By ‘incurable,’ the law likely refers to diseases from which recovery is impossible. However, ‘incurable’ might also be interpreted as ‘chronic’. Today many chronic diseases are manageable, allowing animals to live with them—ranging from leish-maniasis to tumours. In other words, today, nearly everything is treatable, although not everything is resolvable. Euthanasia is reserved for the terminal stages of these diseases, when veterinarians determine that a ‘good death’ is the most humane option for the individual’s well-being because the sufferance caused by the disease is too intense. This is the approach followed by public veterinarians at Muratella, emphasising once again that Muratella is not representative of all of Italy.

The situation becomes even more complex when dealing with behavioural issues, as these are harder to define and even more challenging to address in a shelter environment. However, one distinctive feature of the Italian context stands out in the data from Table 5: euthanasia due to behavioural problems is virtually non-existent. This is the key issue at the heart of the debate we aim to highlight in this paper. It stems from the need to discuss the absence of euthanasia for behavioural problems recorded in our facility. Within the Italian cultural context, this should not be seen as unethical or as weak enforcement of legislative measures, but rather as an expression of the principle that all dogs can be rehabilitated and deserve another chance. However, while this approach is respectable in itself, we believe it warrants discussion, as it raises other challenges (see next paragraph). Last but not least, in this context it is important to discuss the wording ‘of proven dangerousness’. Where is the line that separates a moderately aggressive animal from one of ‘proven dangerousness’? The law does not clarify this, nor does it specify who is to make such a determination and is therefore responsible for it.

### 4.2. The Dark Side of the Moon: The Never-Adopted Dogs

While we acknowledge the valuable work carried out by the shelters (and by Muratella shelter in particular), this paper aims to highlight another aspect of the no-kill policy and shelter management that is rarely considered but deserves a more in-depth discussion. In the context of the ethical debate, information on non-adopted dogs is particularly significant.

As previously mentioned, 307 and 278 dogs remained in the shelter during the two respective five-year periods. Some of these dogs were adopted in later years: 149 (48.53%) from the first period and 82 (29.50%) from the second—including 7 (4.70%) and 8 (9.76%) biting dogs, respectively. This offers hope that more of these dogs may still find homes in the future. However, many others—particularly those displaying aggressive behaviour and considered unapproachable by people or other animals—will likely spend their entire lives in the shelter. Most of these dogs were removed from the streets. However, they were not born free in the territory, nor were their mothers. These are dogs socialised with humans, for whom a stay in a shelter can represent a temporary break from a state of instability to one of safety where food and shelter are available, albeit at the expense of freedom, according to the ‘safe bowl hypothesis’ [22]. Nonetheless, this pause must remain brief; otherwise, the shelter risks losing the original purpose for which it was established under Law no. 281/1991.

Can we really ignore the sad fate of these hundreds of dogs? The number may seem small and insignificant compared to the thousands of dogs adopted out, but we are still talking about hundreds of sentient beings. Can we really ignore this reality, even in the light of the many successful adoptions? This reflection must also be extended to the Italian context, as this study highlights hundreds of dogs spending their entire lives in the Muratella dog shelter. However, the estimated numbers are significantly higher when considering the whole country, particularly the south.

A heated debate has recently emerged, challenging the anthropocentric view that sees the capture of a free-ranging dog from its territory as a ‘rescue action’ and the neutering of free-ranging dogs as a sign of ‘progress’ (e.g., [23,24]).

The rise of these perspectives, after years in which the dominant belief held that nothing is better for a dog than living alongside humans and conforming to their rules, reflects a growing awareness that animals, as sentient beings, have their own interests—interests that sometimes conflict with those of humans [25].

This broader perspective leads to another pressing ethical dilemma: is it justifiable to confine a dog to a shelter for life with no chance of adoption? This, in turn, leads to a fundamental question: is it better to endure a life of suffering or to face a quick, painless death through euthanasia?

This is a question with no definitive answer—not even if we could ask the dogs themselves, as individual variability would make a generalized response impossible. If an answer were to be inferred from this survey (which is not feasible, as Muratella’s numbers cannot be extrapolated to the national level), it would likely lean toward avoiding euthanasia for most dogs. Our analysis indicates that the percentage of dogs with no hope of adoption is low and is limited to truly dangerous animals, diagnosed based on excessive aggressive behaviour not motivated by the context, unpredictability in their actions, and the severity of the injuries caused (however, these characteristics may not be permanent and may change over time). We must bear in mind that “aggressive dogs” and “dangerous dogs” refer to different concepts. Aggressive behaviour has evolved as a means for individuals to compete for essential resources needed for survival, and therefore, all dogs exhibit some degree of aggressiveness. However, persistent aggressive behaviour, especially when occurring out of context, can be considered problematic and may render a dog ‘dangerous’.

Italian national and regional laws do not enforce euthanasia for moderately aggressive dogs or those with chronic but treatable diseases. As a result, the challenge of long-term management arises, along with the associated costs. More importantly, we must consider the quality of life that can be offered to these dogs. Ultimately, in a well-managed shelter, lifelong confinement would appear to be a preferable alternative to euthanasia.

There is a lack of scientific analysis regarding the ethical implications of euthanising healthy but moderately aggressive dogs or sick yet treatable ones. Unfortunately, an even greater issue is the absence of a scientific debate on the ethical concerns surrounding dogs considered dangerous or unapproachable dogs. These individuals, in particular, often suffer from both inter- and intra-specific social deprivation, which can further exacerbate abnormal behaviours [26].

This raises both an ethical dilemma and a management challenge for these animals. These dogs still require care and must be managed in the best possible way to ensure adequate welfare standards. The Length of Stay (LOS) in shelters is closely linked to the likelihood of disease outbreaks and, as mentioned earlier, behavioural deterioration [27]. How can we determine whether a life spent in confinement—deprived of inter- or intra-specific interaction—is more worth living than a humane death? Similar questions were posed in an article on cats, which subtly criticised anthropocentrism and humanity’s tendency to make decisions not only for itself but for all forms of life on Earth [28]. We emphasise the importance of paying close attention to the plight of dogs that remain in shelters but outside the adoption process. Benedetti et al. [29] similarly analysed the situation of aggressive dogs housed in a shelter. In addition to an ethical point of view, they emphasise also the amount of resources that are used when aggressive dogs are involved. The case study reported the improvements of a dangerous dog housed in a shelter, but also underlined the effort (in terms of time and money) that could have been invested in other management strategies or to meet the needs of more, less problematic dogs. Correct management of people and money must be taken into careful consideration, since they are usually limited [30]. So we also ask, proposing an animalistic version of the train dilemma [31]: is it right to exploit so many resources to save a few dangerous individuals with limited results, instead of using the same resources to help many others? Sometimes, however, data support those who advocate for investing in the re-education of problematic dogs housed in shelters. Natoli et al. [22] report that, of the 16 dogs abandoned by their owners in the Muratella shelter for being deemed unmanageable, 10 were later adopted after spending some time there. This was likely a result of some behavioural rehabilitation in the shelter, necessary after poor owner management.

## 5. Conclusions

In conclusion, we propose a more in-depth discussion on the ethical implications of the no-kill policy which condemns thousands of non-adoptable dogs to a life of confinement. This discussion should be enriched and supported by data from other shelters, especially those that lack the management and logistical resources of large adoption centres like “Muratella”. In the southern regions of Italy, for instance, straying represents a much larger issue, and the number of dogs housed in facilities is significantly higher.

Although, as already mentioned, providing solutions is beyond the scope of this paper, one suggestion might be useful: every shelter in Italy should have an interdisciplinary bioethics committee composed of ethologists, veterinarians from the Local Health Unit, the president of the Veterinary Association of the region where the shelter is located, representatives of animal rights organisations, the shelter’s Sanitary Director, and the Director of the Office for Animal Rights of the municipality. Such a committee could help find the best solution for the most complex cases.

As a final point of reflection, we quote a provocative statement from Heinlein [32]: ‘*Perhaps our focus on keeping pets alive, no matter the consequences, is really more about us humans than about a desire to spare animals suffering*’. This perfectly encapsulates our concerns regarding the welfare of animals condemned to live—and die—in shelters.

## Figures and Tables

**Table 1:** The sex, breed, body size and age class of dogs entered the ‘Muratella’ shelter in Rome, Italy, in the first and in the second period.

	First Period	Second Period
Males	4458	4975
Females	3430	3681
Purebreed	1841	2283
Mixed-breed	6047	6373
Puppies (size undetermined)	2843	2536
Body size: small	448	1582
Body size: medium	632	2894
Body size: large	567	1624
Body size: not reported	3398	20
Age class: 0–2 months (0–89 days)	1744	1635
3–4 months (90–149 days)	624	670
5–7 months (150–239 days)	475	514
8 months–1 year (240–364 days)	306	466
1–2 years (365–729 days)	1161	1402
2–5 years (730–1824 days)	1653	1973
5–7 years (1825–2919 days)	910	1033
8–10 years (2920–4014 days)	694	650
11–14 years (4015–5474 days)	293	269
15–20 years (5475–7300 days)	28	44

**Table 2:** Number of individuals adopted and relative percentage for each class (sex, breed, and body size) for dogs adopted, deceased (including natural causes and euthanasia), or still in “Muratella” shelter (Rome, Italy) in the two five-year periods.

Five-Year Period	2012–2016		2018–2022	
	Entered	Adopted	Entered	Adopted
Males	2716	2176 (80.2%)	2645	2189 (82.76%)
Females	2389	2071 (86.69%)	2208	2028 (91.85%)
Purebreed	789	610 (77.31%)	862	670 (77.73%)
Mixed-breed	4316	3637 (84.26%)	3991	3581 (89.73%)
Puppies (size undetermined)	2508	2188 (87.24%)	2127	1980 (93.09%)
Body size: small	159	135 (84.91%)	768	714 (92.97%)
Body size: medium	216	170 (78.70%)	1290	1066 (82.64%)
Body size: large	237	156 (65.82%)	662	486 (73.41%)
Body size: not reported	1985	1598 (80.50%)	6	5 (83.33%)
Age class: 0–2 months (0–89 days)	1581	911 (57.62%)	1407	1171 (83.22%)
3–4 months (90–149 days)	539	428 (79.41%)	568	565 (99.47%)
5–7 months (150–239 days)	388	340 (87.63%)	369	342 (92.68%)
8 months–1 year (240–364 days)	223	440 (197.31%)	290	283 (97.59%)
1–2 years (365–729 days)	681	795 (116.74%)	715	665 (93.01%)
2–5 years (730–1824 days)	787	715 (90.85%)	802	690 (86.03%)
5–7 years (1825–2919 days)	378	315 (83.33%)	359	282 (78.55%)
8–10 years (2920–4014 days)	353	207 (58.64%)	224	175 (78.13%)
11–14 years (4015–5474 days)	159	86 (54.09%)	97	65 (67.01%)
15–20 years (5475–7300 days)	16	10 (62.5%)	22	13 (59.09%)

**Table 3:** Standard error, intercept value, *p*-value, and 95% confidence interval (CI HR) generated with the GLMM for categories of dogs (sex, body size, age class at time of adoption) entered in the shelter in the first period, excluding redeemed or transferred ones (N = 5105). The categories used for comparison (i.e., males, mixed-breed, body size not reported, and 15–20 years) do not report the values of the analysis because they are considered to be redundant.

Variable	Standard Error	Intercept Value	*p*-Value	95% CI HR
Intercept	31.98	363.87	<0.001	[301.18, 426.56]
Females	4.22	−21.11	<0.001	[−29.40, −12.83]
Males	/	/	/	/
Purebreed	11.38	−7.13	=0.531	[−29.44, 15.18]
Mixed-breed	/	/	/	/
Puppies (size undetermined)	9.00	165.26	<0.001	[147.60, 182.92]
Small size	15.38	59.81	<0.001	[29.67, −89.96]
Medium size	15.72	7.29	=0.643	[−23.51, 38.10]
Large size	16.95	−43.35	=0.011	[−76.57, −10.13]
Body size not reported	/	/	/	/
Never adopted	33.07	4591.88	<0.001	[4527.06, 4656.70]
0–2 months (0–89 days)	33.38	−502.77	<0.001	[−568.21, −437.34]
3–4 months (90–149 days)	33.76	−492.02	<0.001	[−558.19, −425.84]
5–7 months (150–239 days)	33.97	−430.71	<0.001	[−497.29, −364.13]
8 months–1 year (240–364 days)	33.30	−340.51	<0.001	[−405.80, −275.23]
1–2 years (365–729 days)	32.33	−223.03	<0.001	[−286.40, −159.66]
2–5 years (730–1824 days)	32.28	−146.40	<0.001	[−209.67, −83.13]
5–7 years (1825–2919 days)	32.78	−160.07	<0.001	[−224.32, −95.82]
8–10 years (2920–4014 days)	32.88	−164.86	<0.001	[−229.30, −100.41]
11–14 years (4015–5474 days)	33.96	−108.20	=0.001	[−174.78, −41.63]
15–20 years (5475–7300 days)	/	/	/	/

**Table 4:** Standard error, intercept value, *p*-value and 95% confidence interval (CI HR) generated with the GLMM for categories of dogs (sex, breed, body size, colour of the coat, age class at time of adoption) entered in the shelter in the second period, excluding redeemed or transferred ones (N = 4853). The categories used for comparison (i.e., males, mixed-breed, body size not reported and 15–20 years) do not report the values of the analysis because they are considered to be redundant.

Variable	Standard Error	Intercept Value	*p* Value	95% CI HR
Intercept	8.51	5027.12	<0.001	[5010.44, 5043.81]
Females	3.42	−18.92	<0.001	[25.63, −12.21]
Males	/	/	/	/
Purebreed	4.33	18.11	<0.001	[9.62, 26.61]
Mixed-breed	/	/	/	/
Puppies (size undetermined)	9.26	−45.32	<0.001	[−63.47, −27.16]
Small size	6.49	−115.50	<0.001	[−128.23, −102.78]
Medium size	5.77	−45.02	<0.001	[−56.35, −33.70]
Large size	/	/	/	/
Light coat colour	4.46	−7.02	=0.116	[−15.79, 1.72]
Mixed coat colour	3.87	0.70	=0.855	[−6.88, 8.29]
Dark coat colour	/	/	/	/
0–2 months (0–89 days)	11.24	−4958.46	<0.001	[−4980.51, −4936.41]
3–4 months (90–149 days)	11.61	−4956.15	<0.001	[−4978.92, −4933.39]
5–7 months (150–239 days)	11.52	4945.74	<0.001	[−4968.33, −4923.15]
8 months–1 year (240–364 days)	10.19	−4926.96	<0.001	[−4946.93, −4906.98]
1–2 years (365–729 days)	8.55	−4902.75	<0.001	[−4919.53, −4885.98]
2–5 years (730–1824 days)	8.42	−4847.35	<0.001	[−4863.85, −4830.84]
5–7 years (1825–2919 days)	9.82	−4818.32	<0.001	[−4837.57, −4799.07]
8–10 years (2920–4014 days)	10.94	−4828.11	<0.001	[−4849.55, −4806.66]
11–14 years (4015–5474 days)	13.86	−4847.81	<0.001	[−4874.99, −4820.63]
15–20 years (5475–7300 days)	24.97	−4924.29	<0.001	[−4973.25, −4875.34]
Never adopted	/	/	/	/

**Table 5:** The number of dogs that entered the shelter already injured or sick, those admitted healthy but subsequently diagnosed with chronic diseases or behavioural problems, as well as those euthanised, deceased, adopted, and not adopted during the first and second periods.

	First Period	Second Period
Euthanised for incurable health problems	147	70
Entered already injured and euthanised	31	9
Entered already ill and euthanised	61	9
Entered already injured and later died naturally	37	21
Entered already ill and later died naturally	94	26
Assisted in the shelter for injuries and adopted	272	126
Assisted in the shelter for illness and adopted	304	85
Assisted in the shelter for injuries and non-adopted	12	6
Assisted in the shelter for illness and non-adopted	27	1
Admitted healthy but subsequently diagnosed with chronic diseases *	data not available	101
Relinquished to the shelter due to various reasons declared by the owner **	303	327
Hosted by the shelter with behavioural problems ***	175	192
Euthanised in the shelter due to proven dangerousness	0	0
Relinquished to the shelter due to behavioural problems declared by the owner	74	104
Rehabilitated in the shelter for behavioural problems and finally adopted	5	51
Rehabilitated in the shelter for behavioural problems but never adopted	13	48

* heart disease, epilepsy, leishmania, Addison’s disease, Cushing’s disease, tumours. ** events of the owner: illness, death, moving to another house and/or town, loss of job, family break-up, or the birth of children in the owner’s household. *** biting dogs.

**Table 6:** The sex, breed, body size and age class of non-adopted dogs in the first and second periods.

	First Period	Second Period
Males	228	218
Females	79	60
Purebreed	72	116
Mixed-breed	236	162
Puppies (size undetermined)	82	1
Body size: small	7	15
Body size: medium	20	153
Body size: large	40	109
Body size: not reported	158	0
Age class: 0–2 months (0–89 days)	56	0
3–4 months (90–149 days)	9	5
5–7 months (150–239 days)	16	9
8 months–1 year (240–364 days)	8	23
1–2 years (365–729 days)	45	53
2–5 years (730–1824 days)	84	105
5–7 years (1825–2919 days)	37	60
8–10 years (2920–4014 days)	32	19
11–14 years (4015–5474 days)	19	4
15–20 years (5475–7300 days)	1	0

**Table 7:** The breed of non-adopted dogs and their status as biters or non-biters during the first and second periods.

Five-Year Period	First Period		Second Period	
	Non-Adopted	Biters	Non-Adopted	Biters
Molossoid breeds and derivatives	34	20 (58.82%)	98	22 (22.45%)
Hunting breeds	8	1 (12.5%)	8	2 (25%)
Herding breeds	27	0	34	6 (17.65%)
Companion breeds	8	0	2	1 (50%)
Search-and-rescue breeds	1	0	0	0
Mixed breeds	228	15 (6.58%)	136	17 (12.5%)
Sledding breeds	1	0	0	0
Total	307	36 (11.73%)	278	48 (17.27%)

**Table 8:** The fate of dogs that were not adopted during the first and second periods, as reviewed during the period from 1 January 2023 to 31 December 2024, and their status as biters or non-biters.

Five-Year Period	First Period Total	First Period Biters	Second Period Total	Second Period Biters
Adopted	149 (48.53%)	7 (4.70%)	82 (29.50%)	8 (9.76%)
Redeemed	3 (0.98%)	0	3 (1.08%)	0
Transferred	125 (40.72%)	53 (42.4%)	66 (23.74%)	11 (16.67%)
Unadopted	12 (3.91%)	0	113 (39.93%)	25 (22.52%)
Deceased *	18 (5.86%)	3 (16.67%)	14 (5.04%)	4 (28.57%)
Total	307	63 (20.52%)	278	48 (17.39%)

* including natural causes and euthanasia.

## Data Availability

The data presented in this study are available on request from the corresponding author.
